# Untargeted Metabolomics Reveals Metabolic Reprogramming Associated with Variable Temperature Stratification During Dormancy Release in *Zanthoxylum armatum* Seeds

**DOI:** 10.3390/biology15090666

**Published:** 2026-04-23

**Authors:** Manyi Fu, Fengjuan Zhou, Chang Liu, Jihong Xiao, Yushan Zheng

**Affiliations:** 1 College of Forestry, Fujian Agriculture and Forestry University, Fuzhou 350002, China; fmy556@xyafu.edu.cn (M.F.);; 2 College of Forestry, Xinyang Agriculture and Forestry University, Xinyang 464000, China

**Keywords:** *Zanthoxylum armatum* DC., seed dormancy release, untargeted metabolomics, variable temperature stratification

## Abstract

The deep physiological dormancy of *Zanthoxylum armatum* DC. seeds severely limits its seedling propagation efficiency. Variable temperature stratification is an effective treatment for promoting dormancy release; however, the metabolic basis underlying this process remains poorly understood. In this study, we utilized a UPLC-MS/MS-based untargeted metabolomics approach, coupled with multivariate statistical analyses (PCA and OPLS-DA), to profile metabolic changes in *Z. armatum* seeds subjected to variable temperature stratification in a moist sand substrate (15 °C in the dark for 10 days, followed by 4 °C for 20 days). A total of 3687 metabolic features were detected, among which 33 structurally annotated differential metabolites were retained for biological interpretation, including 8 upregulated and 25 downregulated metabolites. Pathway enrichment analysis indicated that α-linolenic acid metabolism and linoleic acid metabolism were markedly altered after stratification. In particular, 9-(S)-HPOTE, colneleate, jasmonic acid (JA), and JA-ACC were significantly reduced, suggesting that attenuation of JA-related oxylipin metabolism may be associated with dormancy release in *Z. armatum* seeds. In addition, coordinated changes in phenylpropanoid- and cutin/wax-related metabolites implied remodeling of seed-coat-associated metabolism, whereas the accumulation of branched-chain amino acids and the alteration of sulfur- and purine-related metabolites suggested reorganization of metabolic reserves during the transition from dormancy to germination. Overall, these results provide metabolomic evidence that variable temperature stratification is associated with extensive metabolic reprogramming in *Z. armatum* seeds and highlight JA-related lipid metabolism as a candidate pathway involved in dormancy release.

## Simple Summary

*Zanthoxylum armatum* is an important woody plant used for food and medicine, but its seeds often remain dormant and germinate poorly, which limits seedling production. Although variable temperature treatment is known to improve germination, the internal changes that help seeds wake from dormancy are still not well understood. In this study, we compared untreated seeds with seeds that had received variable temperature treatment and had just started to germinate. We used a broad chemical analysis to examine changes in many small compounds inside the seeds. The results showed that the treatment was linked to major changes in seed chemistry. In particular, several compounds related to plant signaling, seed coat structure, and stored nutrients changed markedly after treatment. These findings suggest that dormancy release in *Zanthoxylum armatum* seeds involves coordinated changes in internal signals, protective outer layers, and nutrient use. This study improves our understanding of why these seeds are difficult to germinate and may help develop more effective methods for raising seedlings in the future.

## 1. Introduction

Seed dormancy serves as a vital adaptive strategy, delaying germination until environmental conditions are favorable to ensure plant survival and population continuity [1]. Yet, for many economically and ecologically valuable species, deep dormancy presents a major bottleneck for artificial propagation and the utilization of germplasm resources [2,3]. While research into dormancy mechanisms has advanced considerably—highlighting the roles of seed coat barriers [4], incomplete embryo development [5], and inhibition by endogenous hormones or secondary metabolites [6]—the specific physiological and biochemical networks orchestrating these processes are still not fully mapped out for many individual species. Complicating matters further is the intricate interplay between environmental cues and genetic factors. The exact molecular mechanisms driving these interactions remain ambiguous, highlighting the need for more targeted, in-depth studies [7].

As a robust systems biology tool, metabolomics has become integral to plant physiological and biochemical research. It enables the tracking of dynamic shifts in small-molecule metabolites across different developmental stages or in response to environmental stimuli [8] and has been widely adopted across various plant species [9,10,11]. Within the genus Zanthoxylum, metabolomic approaches have previously been employed to evaluate how different extraction methods affect volatile and numbing compounds [12], as well as to unravel the formation mechanisms of these numbing substances during fruit development [13]. However, the current literature predominantly focuses on vegetative growth, stress responses, or the broader functions of secondary metabolites. Systematic investigations into the metabolic reprogramming that occurs during treatment-induced seed dormancy release remain scarce. This knowledge gap is particularly evident in economically important crops with complex dormancy mechanisms, where the specific metabolic pathways and key regulatory hubs dictating dormancy release are yet to be fully elucidated.

*Zanthoxylum armatum* DC., an economically important forest tree species, has garnered widespread attention for its distinctive culinary and medicinal properties [14,15]. However, *Z. armatum* seeds typically exhibit deep dormancy, which severely restricts their germination rates and seedling propagation efficiency. This dormancy is primarily driven by two factors: a seed coat rich in waxes and lipids that creates an impermeable mechanical barrier and the requirement for embryo after-ripening coupled with the presence of endogenous inhibitors [16]. Variable temperature stratification, a physical treatment designed to simulate natural environmental cues, is a well-established method for breaking seed dormancy by triggering complex physiological and biochemical responses within the seed [17]. While Phuyal et al. demonstrated the general efficacy of this stratification in breaking the dormancy of Zanthoxylum seeds [18], and similar optimal outcomes have been validated in *Z. schinifolium* [19], the precise molecular mechanisms by which variable temperature stratification modulates the internal metabolism of *Z. armatum* seeds remain elusive. To date, research on *Z. armatum* has predominantly concentrated on chemical profiling [20], biological activity evaluation [21], genomic characterization [22], and quality assessment [23]. Comprehensive analyses detailing the dynamic changes in seed metabolites pre- and post-stratification have not yet been reported.

Accordingly, the present study employed an untargeted metabolomics approach to compare untreated seeds with seeds subjected to variable temperature stratification at the radicle-emergence stage in *Z. armatum*. By integrating UPLC-MS/MS profiling, multivariate statistical analysis, and KEGG pathway annotation, we aimed to identify key differential metabolites and candidate metabolic pathways associated with dormancy release. This work provides a metabolomic framework for understanding the transition from dormancy to germination in *Z. armatum* seeds and offers a basis for future targeted validation of candidate regulatory pathways.

## 2. Materials and Methods

### 2.1. Experimental Materials

Fresh *Z. armatum* fruits were collected from Youyang County, Chongqing, China (108°18′–109°19′ E, 28°19′–29°24′ N). Located in the Wuling Mountains, this region is a primary production area for the species, characterized by a mild and humid climate with an average annual temperature of 13–16 °C. The harvested fruits were shade-dried in the dark for 2–3 days to facilitate the natural separation of the seeds from the pericarps. Subsequently, the seeds were subjected to a 24 h water flotation test; floating, nonviable seeds and debris were discarded, while the fully developed, sunken seeds were collected for downstream experiments.

Randomly sampled seeds were soaked in a 2.5% Na_2_CO_3_ solution (analytical grade, Sinopharm Chemical Reagent Co., Ltd., Shanghai, China) for 12 h. Intermittent stirring with a glass rod was applied during this period to maximize seed contact with the alkaline solution and facilitate defatting. Following surface sterilization with 75% ethanol (Sinopharm Chemical Reagent Co., Ltd., Shanghai, China) for 1 min, the seeds were rinsed three times with sterile water (15–30 s per rinse) and blotted dry with absorbent filter paper. The experiment consisted of two groups: an untreated control group (CK, *n* = 180 seeds) and a treatment group (Change, *n* = 180 seeds) subjected to variable temperature stratification. Upon completion of the treatment, germinating seeds at the radicle-emergence stage (showing visible “whitening” through the seed coat) were collected for metabolomic analysis.

### 2.2. Experimental Methods

#### 2.2.1. Variable Temperature Stratification

River sand was sieved, washed, air-dried, and subsequently autoclaved at 121 °C for 40 min using an autoclave (LDZX-50KBS, Shanghai Shenan Medical Instrument Factory, Shanghai, China) [24,25]. After cooling, sterile distilled water was added to adjust the sand moisture content to approximately 25% (*w*/*w*), corresponding to a moist but non-waterlogged condition in which the sand formed a cohesive ball when squeezed by hand but crumbled upon light touch. During stratification, the moisture status of the sand was checked regularly, and sterile distilled water was added when necessary to maintain this condition. The prepared seeds were mixed with the moist sand and transferred into loosely capped culture flasks for variable temperature stratification, allowing gas exchange while reducing contamination and excessive water loss. In this study, variable temperature stratification refers to seed incubation in a moist sand substrate at 15 °C for 10 d, followed by 4 °C for 20 d. The experiment consisted of six biological replicates per group, with 30 seeds in each replicate. The stratification procedure was conducted as follows: first, the seeds were incubated in an intelligent climate chamber (LGZ-1000B, Hangzhou Lvbo Instrument Co., Ltd., Hangzhou, China) at 15 °C with 60% relative humidity in the dark for 10 d. Subsequently, they were transferred to a 4 °C refrigerator (Haier Biomedical, Qingdao, China) and maintained in the dark for an additional 20 d. For metabolomic analysis, each biological replicate was represented by a pooled sample of 10 seeds. Seeds from the variable temperature stratification group were collected at the radicle-emergence stage after stratification, rinsed with sterile water to remove residual sand, blotted dry, immediately frozen in liquid nitrogen, and stored at −80 °C until metabolite extraction. Control samples were collected from untreated seeds processed in parallel using the same washing, blotting, freezing, and storage procedures, except that they were not subjected to variable temperature stratification.

#### 2.2.2. UPLC-MS/MS Analysis

Chromatographic conditions: Separation was performed on an Agilent 1290 Infinity LC system (Agilent Technologies, Santa Clara, CA, USA) equipped with an ACQUITY UPLC HSS T3 column (2.1 mm × 50 mm, 1.8 µm; Waters Corporation, Milford, MA, USA). The column temperature was maintained at 50 °C, the flow rate was set at 0.3 mL·min^−1^, and the injection volume was 5 µL. The mobile phase consisted of water containing 0.1% formic acid (phase A) and acetonitrile containing 0.1% formic acid (phase B) [26,27]. The total analysis time was 25 min, and the gradient elution program is detailed in Table 1.

Mass spectrometry conditions: Detection was performed on a Q Exactive Plus hybrid quadrupole–Orbitrap mass spectrometer (Thermo Fisher Scientific, Bremen, Germany) equipped with a heated electrospray ionization (HESI) source. The source was operated with spray voltages of 3.0 kV in positive ion mode and −3.0 kV in negative ion mode. To optimize desolvation and ion transmission, the gas flow rates were configured at 40, 16, and 3 arb for the sheath, auxiliary, and sweep gases, respectively. Furthermore, the capillary temperature was maintained at 300 °C, the auxiliary gas heater temperature was set to 350 °C, and the S-lens RF level was kept at 55.

Data were acquired using Xcalibur software (version 4.3, Thermo Fisher Scientific, Waltham, MA, USA) in a data-dependent acquisition (DDA) mode. Full MS scans were performed over a mass range of *m*/*z* 100–1000 at a resolution of 70,000 (at *m*/*z* 200). The automatic gain control (AGC) target and maximum injection time (IT) were configured at 3 × 10^6^ and 100 ms, respectively. For MS/MS analysis, the top 15 most abundant precursor ions were subjected to higher-energy collisional dissociation (HCD). The secondary spectra were acquired at a resolution of 17,500, with an AGC target of 1 × 10^5^, a maximum IT of 50 ms, and an isolation window of 2.0 *m*/*z*. Additionally, stepped normalized collision energies (NCE) were applied at 10, 30, and 50 eV, and the dynamic exclusion duration was set to 4 s.

Data acquisition and processing were performed using Xcalibur software and MS-DIAL software (version 5.0.3, RIKEN Center for Sustainable Resource Science, Yokohama, Japan). Compounds were systematically identified based on their accurate MS1 masses and MS/MS fragmentation patterns. These assignments were further validated through comparisons with reference standards, searches against an in-house database, and diagnostic ion confirmation.

#### 2.2.3. Data Processing

Principal component analysis (PCA) and orthogonal partial least squares discriminant analysis (OPLS-DA) were performed using SIMCA software (version 14.1, Umetrics, Umea, Sweden). PCA was used as an unsupervised method to assess overall sample variation and clustering patterns, whereas OPLS-DA was used as a supervised method to enhance inter-group discrimination by removing orthogonal variation unrelated to classification. Differential metabolites were screened based on the criteria of *p* < 0.05 (*t*-test) and a fold change (FC) ≥ 2 or ≤0.5. Data visualization, comprising volcano plots and heatmaps, was conducted in Origin software (version 2021, OriginLab Corporation, Northampton, MA, USA). Furthermore, key metabolic pathways were identified through enrichment analysis via the KEGG database.

## 3. Results

### 3.1. PCA and OPLS-DA of Z. armatum Seeds Before and After Variable Temperature Stratification

**Figure 1:**
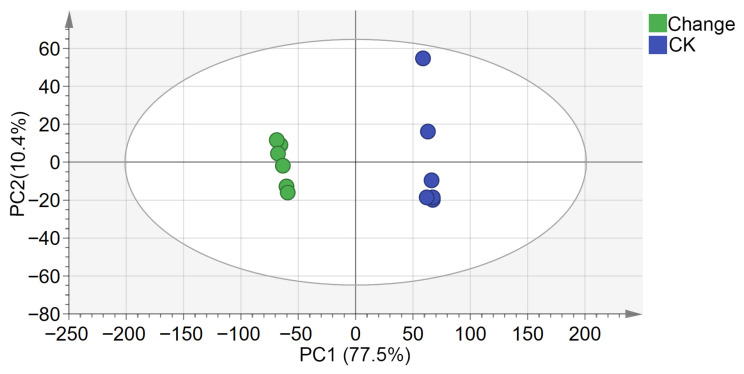
Metabolite PCA score plot of *Z. armatum* seeds before and after variable temperature stratification. CK indicates seeds in the untreated control group (blue scatter points). Change indicates seeds treated with variable temperature stratification (green scatter points).

**Figure 2:**
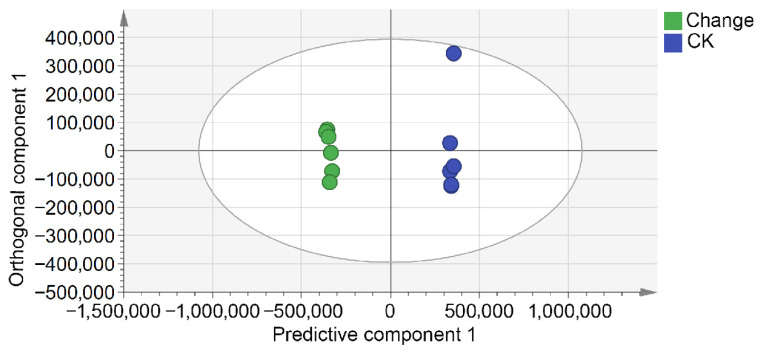
Metabolite OPLS-DA score plot of *Z. armatum* seeds before and after variable temperature stratification. CK indicates the untreated control seeds (blue scatter points). Change indicates the seeds subjected to variable temperature stratification (green scatter points). The distinct separation between the groups indicates that variable temperature stratification was associated with marked differences in the metabolic profile.

The PCA score plot (Figure 1) showed an overall separation trend between CK and Change samples, with biological replicates clustering closely within each group. As depicted in Figure 2, the OPLS-DA score plot demonstrated a clearer separation between the two groups, indicating that the inter-group differences exceeded the intra-group variation.

**Figure 3:**
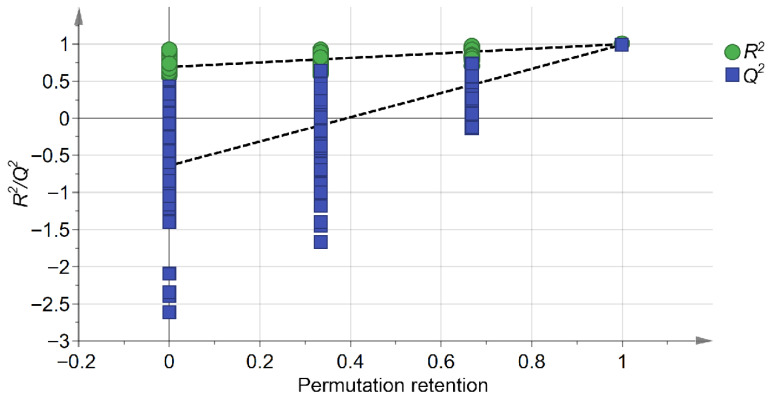
Validation plot of the OPLS-DA model for *Z. armatum* metabolites using a 200-iteration permutation test. Green circles and blue squares indicate the *R*^2^ and *Q*^2^ values, respectively. The dashed lines represent the regression lines of *R*^2^ and *Q*^2^ generated from the permutation test. The y-axis intercepts of the regression lines are *R*^2^ = 0.69 and *Q*^2^ = −0.639, demonstrating the high reliability and robust predictive capacity of the model without overfitting.

Figure 3 presents the 200-iteration permutation test used to validate the robustness of the OPLS-DA model and to assess the risk of overfitting. The validation plot yielded an *R*^2^ intercept of 0.69 and a *Q*^2^ intercept of −0.639, indicating that the model was stable and showed no evident overfitting. Thus, Figure 2 and Figure 3 provide complementary information, representing group discrimination and model validation, respectively. Overall, both PCA and OPLS-DA suggested that variable temperature stratification was associated with marked changes in the endogenous metabolite profile of *Z. armatum* seeds.

### 3.2. Differential Metabolites in Z. armatum Seeds Before and After Variable Temperature Stratification

**Figure 4:**
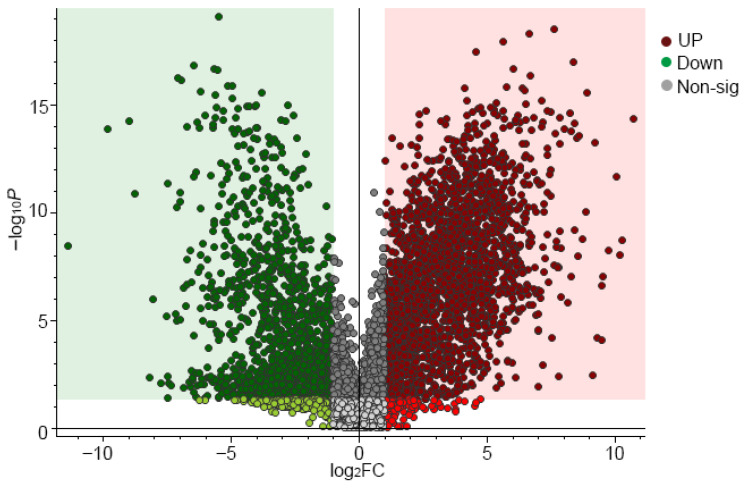
Volcano plot of differential metabolites in *Z. armatum* seeds before and after variable temperature stratification. The x- and y-axes display the log_2_ fold change (log_2_FC) in relative metabolite abundance and the statistical significance level (−log_10_ *p*), respectively. Red, green, and grey scatter points denote significantly upregulated, downregulated, and non-significantly changed metabolites following the treatment, respectively (screening thresholds: *p* < 0.05 and |log_2_FC| ≥ 1).

To elucidate the metabolic regulatory mechanisms triggered by variable temperature stratification in *Z. armatum* seeds, the differential metabolites before and after the treatment were systematically screened. A volcano plot was generated to concurrently visualize the magnitude (log_2_ fold change, x-axis) and statistical significance (−log_10_ *p*-value, y-axis) of the altered metabolite abundances. Applying the established thresholds of *p* < 0.05 and |log_2_FC| ≥ 1, the red and green scatter points denote differential metabolic features that were significantly upregulated and downregulated, respectively, following stratification (Figure 4).

Through the untargeted metabolomics approach, a total of 3687 metabolic features were detected between the control (CK) and variable temperature stratification (Change) groups. Applying the strict significance thresholds (*p* < 0.05 and |log_2_FC| ≥ 1) yielded a preliminary set of differential metabolites. To guarantee the biological reliability of the findings, features lacking definitive annotations, potential exogenous contaminants, and non-plant-derived artifacts were rigorously filtered out. Consequently, 33 key differential metabolites with unambiguous structural information were successfully identified (Table 2). Of these, the relative abundances of 8 metabolites were significantly increased, whereas 25 were significantly decreased following the stratification treatment.

According to their chemical structures and biological functions, the 33 key differential metabolites were categorized into seven major classes, comprising 10 lipids, fatty acids, and derivatives; 6 terpenoids and other compounds; 5 amino acids and their derivatives; 4 phenylpropanoids and phenolic acids; 3 phytohormones and alkaloids; 3 nucleosides; and 2 organic acids. Overall, the variable temperature stratification induced a targeted reorganization of the internal metabolic network within the seeds. Notably, phytohormones and alkaloids, nucleosides, as well as phenylpropanoids and phenolic acids were universally downregulated following stratification. Similarly, decreased abundances dominated the lipid (7 downregulated) and amino acid (4 downregulated) categories. In contrast, organic acids participating in primary metabolism consistently accumulated, exhibiting significant upregulation (Table 3).

### 3.3. Hierarchical Clustering Analysis of Differential Metabolites in Z. armatum Seeds Before and After Variable Temperature Stratification

**Figure 5:**
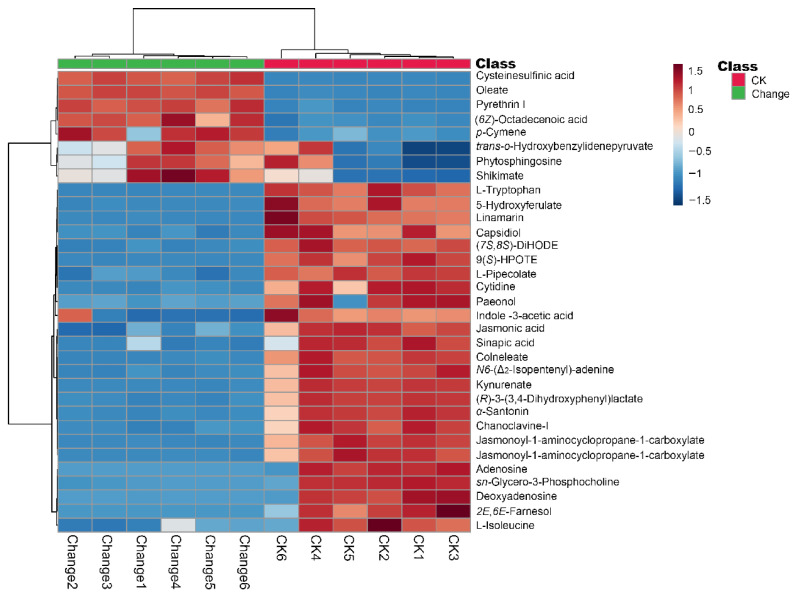
Hierarchical clustering heatmap of differential metabolites in *Z. armatum* seeds before and after variable temperature stratification. The x- and y-axes display the biological replicates and the 33 differential metabolites, respectively. The color scale indicates the normalized relative abundances (Z-scores), where red and blue denote high and low abundance levels, respectively. The dendrograms illustrate the hierarchical clustering of samples and metabolites based on their abundance similarities. CK and Change indicate the untreated control and variable temperature stratification groups, respectively.

Hierarchical clustering was performed on the 33 key differential metabolites to visualize their abundance patterns across CK and Change samples and to explore their biological relevance during variable temperature stratification (Figure 5). In the heatmap, the color scale represents normalized metabolite abundance, with red and blue indicating relatively high and low abundance, respectively. Because the separation between CK and Change samples had already been demonstrated by PCA and OPLS-DA, the interpretation below focuses mainly on the biological significance of the two metabolite clusters.

Cluster I contained most of the differential metabolites, including phytohormones, nucleosides, lipids, and several phenylpropanoid-related compounds. These metabolites showed relatively high abundance in CK seeds but decreased after variable temperature stratification, suggesting a reduction in metabolites associated with hormonal regulation, storage compound turnover, and secondary metabolic processes during dormancy release. Conversely, Cluster II included a smaller group of metabolites, such as organic acids, selected terpenoids, and oleate-related compounds, that accumulated in the Change group. The increased abundance of these metabolites may reflect the activation of selected primary metabolic and lipid-related processes during the transition from dormancy to early germination. Overall, the two clusters indicate that variable temperature stratification was associated with coordinated decreases in several dormancy-related or reserve-associated metabolites and increases in selected metabolites potentially linked to early germination metabolism.

### 3.4. KEGG Pathway Enrichment Analysis of Z. armatum Seeds Before and After Variable Temperature Stratification

**Figure 6:**
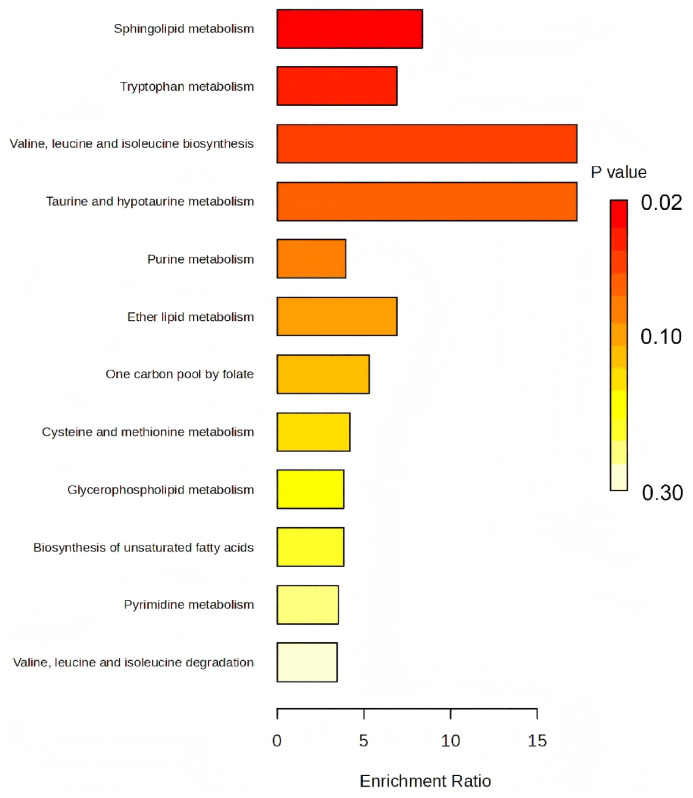
KEGG pathway enrichment analysis of differential metabolites in *Z. armatum* seeds before and after variable temperature stratification. The x- and y-axes display the enrichment ratio and the corresponding KEGG pathway names, respectively. The color gradient of the bars (from yellow to red) indicates increasing statistical significance (i.e., decreasing *p*-values).

To systematically elucidate the molecular regulatory networks governing dormancy release in *Z. armatum* seeds, KEGG pathway enrichment analysis was performed on the differential metabolites (Figure 6). The metabolic reprogramming induced by the variable temperature stratification followed two distinct enrichment trajectories.

The first trajectory was a “high enrichment ratio” pattern, predominantly associated with material storage and mobilization. Specifically, the pathways for valine, leucine, and isoleucine biosynthesis, alongside taurine and hypotaurine metabolism, exhibited exceptionally high enrichment ratios. This pronounced enrichment suggests that robust de novo amino acid synthesis and conversion occurred during stratification, establishing a substantial pool of free branched-chain amino acids to fuel the initiation of germination. Concurrently, the significant upregulation of cysteinesulfinic acid (Table 2) points to the activation of sulfur metabolism, potentially bolstering the seeds’ stress resistance and antioxidant capacity during the cold phase of stratification.

The second trajectory was a “high significance” pattern, characterized by core signal regulation. While sphingolipid metabolism and tryptophan metabolism displayed moderate enrichment ratios compared to the aforementioned pathways, their statistical significance (*p*-values) ranked at the forefront. Such remarkable significance implies that, rather than merely supporting basal metabolism, these pathways exert a central regulatory function in the signaling cascades that orchestrate dormancy release.

**Figure 7:**
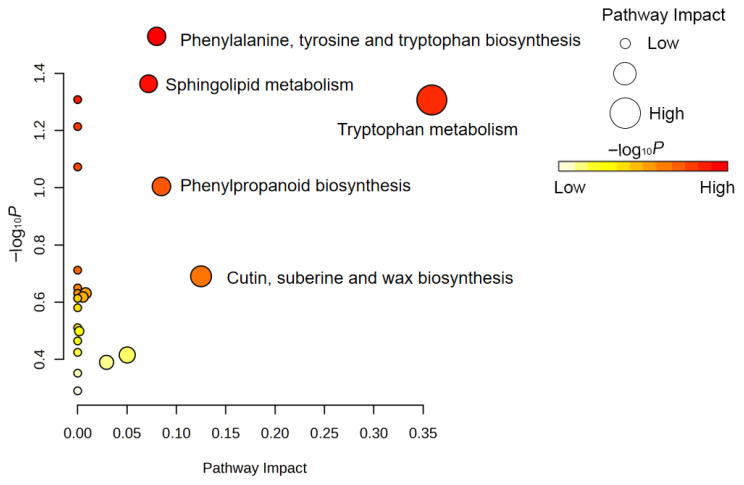
Bubble plot of KEGG pathway topology analysis for differential metabolites in *Z. armatum* seeds before and after variable temperature stratification. The x-axis (Pathway Impact) and y-axis (−log_10_ *p*) display the impact values of key nodes within the pathways and the statistical significance of the enrichment, respectively. Bubble size positively correlates with the pathway impact, whereas the color gradient (from yellow to red) indicates decreasing *p*-values (i.e., increasing significance).

To further validate these speculations and pinpoint the core regulatory hubs, a pathway topology analysis based on network centrality was introduced (Figure 7). This approach identified key metabolic nodes governing dormancy release through a dual-dimensional evaluation of statistical significance (y-axis) and pathway impact (x-axis). Notably, tryptophan metabolism was positioned at the far right of the bubble plot, exhibiting the highest pathway impact value. As a crucial precursor pathway for auxin (IAA) biosynthesis, the pronounced shift in tryptophan metabolic flux suggests that variable temperature stratification precisely targets the endogenous hormonal network, thereby triggering the signaling cascades necessary for dormancy breaking.

Concurrently, phenylalanine, tyrosine, and tryptophan biosynthesis, located at the top of the topology plot, displayed extreme statistical significance. Serving as the upstream synthetic hub for aromatic amino acids, the activation of this pathway plays a vital metabolic routing role: it not only supplies precursors for the downstream synthesis of tryptophan and IAA but also provides the carbon skeletons for phenylpropanoid secondary metabolism. This interpretation is also supported by the enrichment of phenylpropanoid biosynthesis together with cutin, suberine, and wax biosynthesis. These pathways are associated with phenylpropanoid-derived cell wall components and surface lipid-related metabolism. However, because no direct anatomical observation or permeability assay was performed in the present study, these results should be interpreted as evidence of seed-coat-associated metabolic changes rather than direct proof of structural remodeling. Therefore, the enrichment of these pathways may suggest potential metabolic remodeling of seed coat barrier-related components during stratification, but further physical and anatomical evidence is required to confirm whether seed coat permeability was actually altered. Furthermore, the remarkable significance of sphingolipid metabolism indicates potential membrane-associated lipid remodeling in response to temperature fluctuations.

Altogether, the integration of KEGG enrichment and topology analyses suggests that variable temperature stratification is associated with multidimensional metabolic reprogramming during dormancy release in *Z. armatum* seeds. The enrichment of branched-chain amino acid, purine, and sulfur metabolism indicates coordinated changes in reserve mobilization, energy-related metabolism, and stress-response processes. In addition, aromatic amino acid biosynthesis may act as an upstream metabolic hub linked to two downstream trends: one associated with hormonal and membrane-related metabolism through tryptophan and sphingolipid pathways, and the other associated with potential changes in seed-coat-related metabolism through phenylpropanoid and wax-associated pathways.

### 3.5. Alterations in α-Linolenic Acid- and Linoleic Acid-Related Metabolites During Variable Temperature Stratification in Z. armatum Seeds

**Figure 8:**
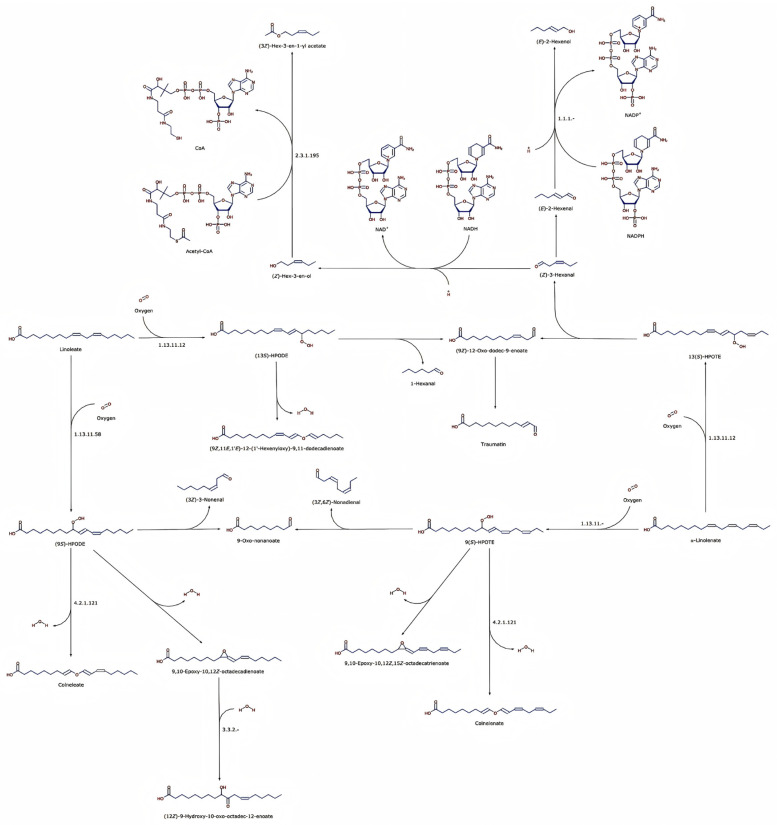
Lipoxygenase (LOX)-mediated metabolic network of α-linolenic acid and linoleic acid in *Z. armatum* seeds. The diagram illustrates the oxidative cascade of free polyunsaturated fatty acids catalyzed by key enzymes (e.g., LOX, EC 1.13.11.12). Specifically, the (**left**) branch delineates the metabolic flux from linoleate to products such as colneleate, whereas the (**right**) branch maps the progression from α-linolenate to 9-(S)-HPOTE and downstream oxylipins. Arrows indicate the direction of metabolic conversion. The colors in the chemical structures follow standard chemical drawing conventions.

Integration of the KEGG enrichment analysis and differential metabolite screening indicated marked changes in lipid-related metabolites, particularly those associated with α-linolenic acid and linoleic acid metabolism, during variable temperature stratification in *Z. armatum* seeds (Figure 8). Figure 8 summarizes the metabolic relationships through which polyunsaturated fatty acids are linked to oxylipin- and phytohormone-related metabolites via LOX-associated pathways. In this context, several metabolites in both the linoleic acid and α-linolenic acid branches showed coordinated decreases after stratification.

In the linoleic acid branch (Figure 8, left), the relative abundance of colneleate—a downstream product of linoleic acid oxidation—decreased significantly following variable temperature stratification (Table 2). Since colneleate acts as a divinyl ether-type plant defense compound, its reduction implies a gradual attenuation of linoleic acid peroxidation-induced defense responses within the seeds. Parallelly, in the α-linolenic acid branch (Figure 8, right), the pivotal intermediate 9-(S)-HPOTE was also markedly downregulated (Table 2). This finding is paramount, as the α-linolenic acid pathway serves as the core biosynthetic route for jasmonic acid (JA), a critical endogenous phytohormone. Indeed, data from Table 2 confirm that both JA (the terminal product) and its amino acid conjugate, JA-ACC, experienced highly consistent and significant downregulation.

**Figure 9:**
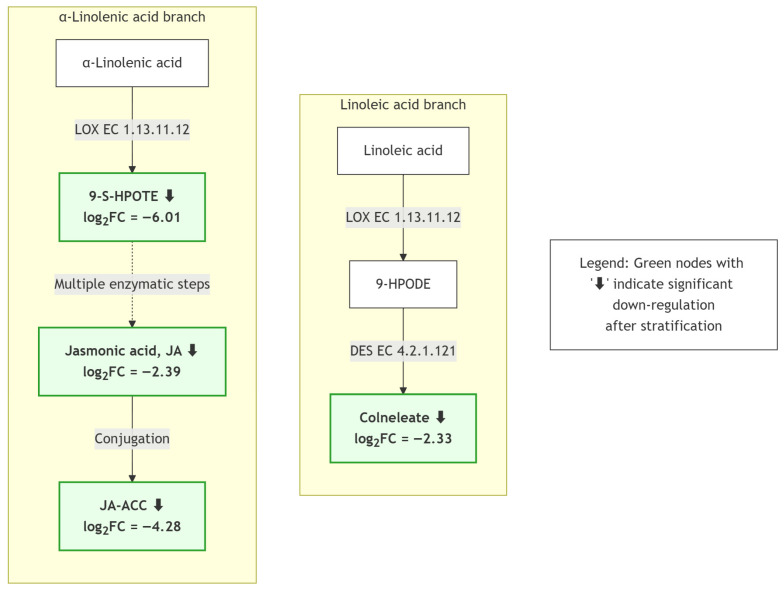
Simplified schematic summary of the core differential metabolic responses within the LOX-related pathway during variable temperature stratification in *Z. armatum* seeds. The detected metabolites identified in this study are directly mapped onto the schematic. Green nodes with downward arrows indicate significantly downregulated metabolites, and the adjacent values represent log_2_ fold change (Change vs. CK).

To more directly visualize the detected differential metabolites within the LOX-related pathway, a simplified schematic summary of the key responsive nodes was generated (Figure 9). In this figure, the detected metabolites are mapped directly onto the schematic, and their downregulation is indicated together with the corresponding log_2_ fold change values. Because the present study is based on untargeted metabolomics rather than direct enzymatic or flux measurements, Figure 9 should be interpreted as a hypothesis-generating model. The observed decreases in colneleate, 9-(S)-HPOTE, JA, and JA-ACC may reflect reduced JA-related oxylipin metabolism during stratification, but further functional analyses are required to determine whether LOX activity or JA biosynthetic flux was directly affected.

## 4. Discussion

The present study shows that variable temperature stratification was associated with extensive metabolic reprogramming in *Z. armatum* seeds. The most prominent pattern was the coordinated decrease in several metabolites related to α-linolenic acid- and linoleic acid-derived oxylipin metabolism, including 9-(S)-HPOTE, colneleate, JA, and JA-ACC. Because these compounds are functionally linked to JA biosynthesis and signaling, their decline suggests that attenuation of JA-related oxylipin metabolism may accompany the transition from dormancy to germination. Although the balance between abscisic acid (ABA) and gibberellin (GA) is conventionally regarded as the classical model for dormancy regulation [6], ABA and GA were not quantified in the present study. Therefore, our data do not allow a direct comparison of the relative importance of JA-related metabolism and the ABA/GA axis. Instead, the reduction in JA-related metabolites should be interpreted as a prominent metabolic feature concurrent with dormancy release in *Z. armatum* seeds. Further targeted hormone quantification and functional validation are needed to clarify how JA-related oxylipin metabolism interacts with ABA/GA signaling during this process. Notably, the abundance of 9-(S)-HPOTE, an intermediate in the α-linolenic acid branch and a known precursor related to JA biosynthesis [28], decreased substantially following stratification. Concurrently, colneleate, originating from the linoleic acid branch, also showed a consistent decrease. These results suggest that variable temperature stratification may be associated with reduced LOX-related oxylipin metabolism or altered substrate flux toward peroxidation. Consequently, the observed reduction in JA-related metabolites may reflect attenuation of oxylipin signaling during dormancy release, rather than direct proof of suppressed LOX activity or blocked JA biosynthesis. Further enzymatic and functional analyses are required to test this hypothesis. More broadly, reduced oxylipin signaling may represent one possible metabolic feature associated with how deeply dormant seeds respond to temperature cues, although this possibility remains to be evaluated in other species.

Beyond the attenuation of signaling pathways, variable temperature stratification significantly alters the internal material reserves and energy metabolism of the seeds. Our data revealed a substantial enrichment of the valine, leucine, and isoleucine biosynthesis pathways (branched-chain amino acids, BCAAs), coupled with a marked depletion of free nucleosides. This metabolic profile aligns with the findings of Zhang et al. [24] regarding storage protein degradation and amino acid accumulation during stratification. The accumulation of BCAAs not only supplies essential raw materials for protein synthesis following germination induction but may also serve as critical signaling molecules facilitating the cell cycle transition from the G1 to the S phase [29]. Concurrently, the enhancement of sulfur metabolism—highlighted by the significant upregulation of cysteinesulfinic acid—activates the antioxidant defense system. This response mitigates oxidative stress induced by the cold phase of stratification, thereby protecting the embryo from excessive reactive oxygen species (ROS) damage. Furthermore, the reduction in adenosine and deoxyadenosine within purine metabolism indicates that free nucleosides are being rapidly consumed for ATP and nucleic acid synthesis. This provides a robust, energetic and genetic foundation for the developmental shift from dormancy to germination [25].

Restricted water and gas permeability of the seed coat constitutes the primary physical barrier imposing deep dormancy in *Z. armatum* seeds [16]. In this study, the enrichment of phenylpropanoid biosynthesis and cutin, suberine, and wax biosynthesis pathways, together with changes in related metabolites, suggests that seed-coat-associated metabolism may be affected by variable temperature stratification. Because these pathways are commonly related to cell wall properties and hydrophobic surface barriers, we hypothesize that such metabolic changes may be associated with potential remodeling of seed coat barrier components. However, this hypothesis requires further validation because no direct seed coat permeability assay, microscopic anatomical observation, or targeted analysis of seed coat structural components was performed in the present study. Future studies should combine metabolomics with physical and anatomical evidence to determine whether these metabolic changes are accompanied by actual changes in seed coat structure and permeability.

Future studies should focus on the functional validation of JA-related oxylipin metabolism during variable temperature stratification. In particular, targeted quantification of JA-related metabolites, combined with perturbation experiments using JA biosynthesis inhibitors or antagonists during stratification, may help determine whether the observed attenuation of JA-related metabolism is causally involved in dormancy release in *Z. armatum* seeds.

## 5. Conclusions

In summary, untargeted metabolomics revealed that variable temperature stratification was associated with pronounced metabolic reprogramming in *Zanthoxylum armatum* seeds. The coordinated reduction in 9-(S)-HPOTE, colneleate, JA, and JA-ACC suggests that attenuation of JA-related oxylipin metabolism was a prominent metabolic feature concurrent with dormancy release, while changes in amino acid-, sulfur-, purine-, phenylpropanoid-, and cutin/wax-related metabolites indicate broader reorganization of reserve metabolism and seed-coat-associated processes. These findings provide a metabolomic basis for understanding dormancy release in *Z. armatum* and identify JA-related lipid metabolism as a candidate pathway for future targeted validation.

## Figures and Tables

**Table 1:** Chromatographic gradient elution program.

Time (min)	Flow Rate (mL·min^−1^)	Mobile Phase A (%)	Mobile Phase B (%)
0	0.3	98	2
1	0.3	98	2
18	0.3	0	100
22	0.3	0	100
22.1	0.3	98	2
25	0.3	98	2

**Table 2:** List of the 33 differential metabolites screened in *Z. armatum* seeds.

Category	Metabolite Name	Formula	*m*/*z*	*p*-Value	log_2_FC	Trend
Lipids, fatty acids and derivatives	Jasmonic acid	C_12_H_18_O_3_	211.13396	3.43 × 10^−5^	−2.39	Down
	Jasmonoyl-1-aminocyclopropane-1-carboxylate	C_16_H_23_NO_4_	294.17159	3.25 × 10^−14^	−4.28	Down
	9(*S*)-HPOTE	C_18_H_30_O_4_	311.22326	3.12 × 10^−10^	−6.01	Down
	(*7S*,*8S*)-DiHODE	C_18_H_32_O_4_	313.23885	7.22 × 10^−9^	−2.32	Down
	Colneleate	C_18_H_30_O_3_	277.21766	5.55 × 10^−6^	−2.33	Down
	(*6Z*)-Octadecenoic acid	C_18_H_34_O_2_	283.26468	7.00 × 10^−5^	3.22	Up
	Oleate	C_18_H_34_O_2_	283.26469	7.96 × 10^−10^	2.86	Up
	Phytosphingosine	C_18_H_39_NO_3_	318.30181	0.047	2.55	Up
	3-Dehydrosphinganine	C_18_H_37_NO_2_	300.29127	1.36 × 10^−4^	−2.71	Down
	*sn*-Glycero-3-Phosphocholine	C_8_H_20_NO_6_P	275.13643	0.001	−3.53	Down
Phenylpropanoids and phenolic acids	Paeonol	C_9_H_10_O_3_	149.06063	0.005	−3.68	Down
	Sinapic acid	C_11_H_12_O_5_	242.10331	9.89 × 10^−10^	−3.91	Down
	5-Hydroxyferulate	C_10_H_10_O_5_	211.06106	2.04 × 10^−10^	−3.02	Down
	(*R*)-3-(3,4-Dihydroxyphenyl)lactate	C_9_H_10_O_5_	181.05059	2.37 × 10^−9^	−3.35	Down
Amino acids and derivatives	L-Tryptophan	C_11_H_12_N_2_O_2_	188.07165	6.72 × 10^−12^	−3.22	Down
	L-Isoleucine	C_6_H_13_NO_2_	132.1026	0.002	−2.03	Down
	Kynurenate	C_10_H_7_NO_3_	190.05097	2.52 × 10^−9^	−2.48	Down
	Cysteinesulfinic acid	C_3_H_7_NO_4_S	154.01691	1.16 × 10^−15^	4.07	Up
	L-Pipecolate	C_6_H_11_NO_2_	130.08714	1.85 × 10^−7^	−2.67	Down
Organic acids	Shikimate	C_7_H_10_O_5_	157.05041	0.012	3.85	Up
	*trans*-*o*-Hydroxybenzylidenepyruvate	C_10_H_8_O_4_	193.05061	0.046	2.18	Up
Phytohormones and alkaloids	Indole-3-acetic acid	C_10_H_9_NO_2_	176.07154	1.46 × 10^−4^	−2.03	Down
	*N*6-(Δ^2^-Isopentenyl)-adenine	C_10_H_13_N_5_	204.12422	1.20 × 10^−7^	−5.95	Down
	Chanoclavine-I	C_16_H_20_N_2_O	256.06063	3.13 × 10^−9^	−3.60	Down
Terpenoids and other compounds	*p*-Cymene	C_10_H_14_	135.11764	1.04 × 10^−4^	2.34	Up
	*2E*,*6E*-Farnesol	C_15_H_26_O	223.20669	9.78 × 10^−9^	−10.19	Down
	Capsidiol	C_15_H_24_O_2_	237.18604	4.27 × 10^−6^	−3.04	Down
	Pyrethrin I	C_21_H_28_O_3_	329.21059	7.44 × 10^−6^	4.60	Up
	α-Santonin	C_15_H_18_O_3_	247.1342	2.78 × 10^−8^	−2.24	Down
	Linamarin	C_10_H_17_NO_6_	248.11406	4.05 × 10^−13^	−5.17	Down
Nucleosides	Cytidine	C_9_H_13_N_3_O_5_	244.09409	0.010	−2.92	Down
	Adenosine	C_10_H_13_N_5_O_4_	268.10545	0.011	−4.43	Down
	Deoxyadenosine	C_10_H_13_N_5_O_3_	252.11035	0.005	−6.12	Down

**Table 3:** Classification and abundance trends of the differential metabolites.

Category	Upregulated	Downregulated
Lipids, fatty acids, and derivatives	3	7
Phenylpropanoids and phenolic acids	0	4
Amino acids and derivatives	1	4
Organic acids	2	0
Phytohormones and alkaloids	0	3
Terpenoids and other compounds	2	4
Nucleosides	0	3

## Data Availability

The raw data supporting the conclusions of this article will be made available by the authors on request.
